# Single-cell and spatially resolved analysis uncovers cell heterogeneity of breast cancer

**DOI:** 10.1186/s13045-022-01236-0

**Published:** 2022-03-03

**Authors:** Si-Qing Liu, Zhi-Jie Gao, Juan Wu, Hong-Mei Zheng, Bei Li, Si Sun, Xiang-Yu Meng, Qi Wu

**Affiliations:** 1 Department of Breast and Thyroid Surgery, Renmin Hospital of Wuhan University, Wuhan, Hubei People’s Republic of China; 2 Department of Pathology, Renmin Hospital of Wuhan University, Wuhan, Hubei People’s Republic of China; 3 Department of Breast Surgery, Hubei Cancer Hospital, Tongji Medical College, Huazhong University of Science and Technology, Wuhan, Hubei People’s Republic of China; 4 Department of Clinical Laboratory, Renmin Hospital of Wuhan University, Hubei Province, 238 Ziyang Road, Wuhan, 430060 People’s Republic of China; 5 Center for Single-Cell Omics and Tumor Liquid Biopsy, Zhongnan Hospital of Wuhan University, Wuhan, Hubei People’s Republic of China; 6 Tongji University Cancer Center, Tenth People’s Hospital of Tongji University, Shanghai, People’s Republic of China

**Keywords:** Single-nucleus RNA sequencing, Spatial transcriptomics, Breast cancer, Tissue architecture, Heterogeneity

## Abstract

The heterogeneity and the complex cellular architecture have a crucial effect on breast cancer progression and response to treatment. However, deciphering the neoplastic subtypes and their spatial organization is still challenging. Here, we combine single-nucleus RNA sequencing (snRNA-seq) with a microarray-based spatial transcriptomics (ST) to identify cell populations and their spatial distribution in breast cancer tissues. Malignant cells are clustered into distinct subpopulations. These cell clusters not only have diverse features, origins and functions, but also emerge to the crosstalk within subtypes. Furthermore, we find that these subclusters are mapped in distinct tissue regions, where discrepant enrichment of stromal cell types are observed. We also inferred the abundance of these tumorous subpopulations by deconvolution of large breast cancer RNA-seq cohorts, revealing differential association with patient survival and therapeutic response. Our study provides a novel insight for the cellular architecture of breast cancer and potential therapeutic strategies.

**Supplementary Information:**

The online version contains supplementary material available at 10.1186/s13045-022-01236-0.

To the editor,

A universal feature of breast cancer (BC) is the complex cellular ecosystems, whereby intra- and inter-tumor heterogeneity is crucial for determining malignant progression and response to treatment. In this study, we aimed to decipher the heterogeneity and complex architecture of breast cancer by combining two complementary high-resolution omics tools [1, 2].

We analyzed 2 primary tumors (BC-A and BC-B) by single-nucleus RNA sequencing (snRNA-seq, 10 × genomics) [2, 3]. Fresh samples from BC-A were analyzed in parallel by spatial transcriptomics (ST, 10 × genomics) (Fig. 1a) [4, 5]. By analyzing the transcriptomes, we detected 9 distinct cell clusters in the BC-A tumors by using canonical lineage markers (Fig. 1b). The epithelial cells were further re-clustered, and 6 subclusters were identified, respectively (Fig. 1c). The copy number variation (CNV) profiles of the BC-A tumor cells show a substantial degree of heterogeneity, suggesting that the origin of these subclusters was variable. Chr8q gain in genomic unstable luminal-A cells was found in the BC-A profile (Additional file 1: Figure S1A). Furthermore, the malignant subclusters were annotated with cluster-specific genes mainly including the basal subtype (*EGFR*^+^*KIT*^+^ basal cells and *EGFR*^+^*TP63*^+^ normal-like cells) and luminal subtype (cycling luminal-B cells, *ERBB4*^+^ luminal-A cells, *ERBB4*^+^*AREG*^+^ luminal-A cells and genomic unstable luminal-A cells) in the BC-A sample (Fig. 1c–e) (Additional file 2: Result S1). To characterize the functional features of these distinct cancer clusters, we dissected their differentially expressed genes. Cycling luminal-B (LumB) cells were uniquely enriched for hallmarks of the cell cycle and proliferation (e.g., E2F and MYC TARGETS), and this cluster exhibited a high metabolic state with the activation of glycatabolism, fatty acid and cholesterol metabolism potentially regulated by PI3K/AKT/mTOR signaling, hypoxia and MYC (Fig. 1f, Additional file 1: Figure S1B-C). In contrast, genomic unstable luminal-A (LumA) cells only showed an activated KRAS-downregulated signaling (Fig. 1f). Next, we asked whether transcription factors (TFs) contribute to the phenotypic state of these subpopulations (Fig. 1g). First, GATA3, a key mediator during the differentiation from luminal progenitor to mature luminal cells, was active in these luminal clusters. AP-1 transcription factor complexes, including *JUN*, *JUNB*, *JUND*, *FOS* and *FOSB*, were enriched in *ERBB4*^+^*AREG*^+^ LumA, genomic unstable LumA, *EGFR*^+^*KIT*^+^ basal and cycling LumB clusters. Likewise, *ERBB4*^+^*AREG*^+^LumA cluster also highly expressed SREBF1/2 which are crucial for controlling cholesterol homeostasis [6]. Pseudotime analysis revealed trajectories of luminal and basal/myoepithelial lineages (Fig. 1h). The six major cell clusters were corroborated by typical mammary lineage marker genes for basal progenitor/myoepithelium (e.g., *MYLK* and *ACTA2*), mature luminal cells (e.g., *ESR1*, *FOXA1*, *PGR* and *AFF3*) and luminal progenitor cells (e.g., *EZH2*) (Additional file 1: Figure S3C-D) [7]. Moreover, the myoepithelial cells stemmed from basal progenitor cells, while luminal progenitor cells gradually differentiated into mature luminal cells (Fig. 1h). Interestingly, a cluster of luminal cells expressed certain level of basal-like genes, suggesting that they may emerge as a dedifferentiated state or represent invasiveness. *EGFR*^+^*TP63*^+^ normal-like subtype was fully distributed in the left arm, while cycling LumB cluster was completely situated in the arm of the luminal progenitor (Fig. 1h). Then, we investigated the interactions between ligands and receptors across all neoplastic populations. Interestingly, *ERBB4*^+^*AREG*^+^ LumA cells secreted increasing EGF and EGF-like cytokine-like AREG, which could combine with EGF receptors such as *EGFR* and *ERBB4* (Fig. 1i, Additional file 1: Figure S1D) (Additional file 2: Result S2).

**Fig. 1:**
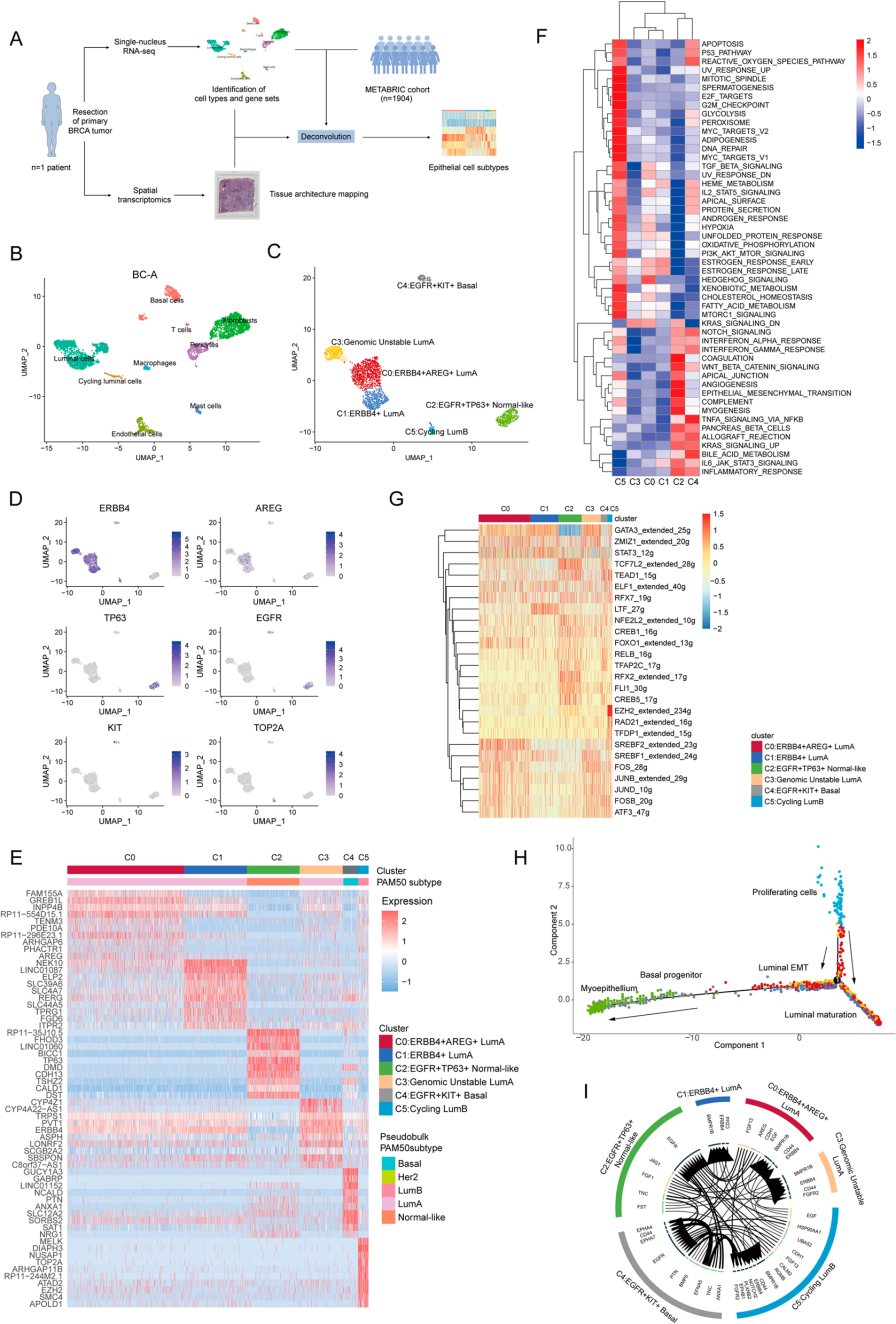
snRNA-seq analysis of tumor from sample BC-A. **a** Schematic of the single-nucleus RNA-seq and ST experiment and analysis. **b** UMAP visualization of 4,093 nuclei from BC-A tumor analyzed by snRNA-seq showing nine major cell types. **c** UMAP visualization of inferred epithelial cells from BC-A tumor analyzed by snRNA-seq. Clusters are colored and labeled according to their inferred cell subtypes. **d** Feature plot of subcluster-specific marker genes in epithelial cells. **e** Heatmap of differentially expressed genes in each epithelial subcluster. The color bars above the heatmap reflects the subcluster PAM50 subtype estimated by ‘pseudobulk.’ **f** Heatmap showing the pathway enrichment of each epithelial subcluster using MSigDB HALLMARK gene sets. Mean score of GSVA was z-score transformed. **g** Heatmap of the area under the curve (AUC) scores of TF motifs estimated per cell by SCENIC. **h** Differentiation trajectories of epithelial cells by Monocle 2. **i** Circos plot showing the interactions between ligands and receptors across cell types

Subsequently, we sought to annotate the intratumoral heterogeneity by integrating the snRNA-seq and ST datasets [5]. The ST dataset was classified into six main areas: the luminal region, basal region, the interfacing area between the luminal region and basal region, stroma and infiltrating lymphocyte areas based on the principal component scores across all ST spots (Fig. 2a, b, Additional file 1: S5C). We found that these luminal subtypes were principally assembled in the luminal region and the interfacing area except that the cycling LumB cells were scattered throughout the tissue. Interestingly, the *ERBB4*^+^ LumA type scarcely existed in the interfacing area (Fig. 2a, b, Additional file 1: S5B). Finally, the spatial division of the primary nontumor cells was identified in the ST dataset, showing that neutrophils were enriched in the luminal region, while B cells principally infiltrated into the basal region (Fig. 2c) (Additional file 2: Result S3).

**Fig. 2:**
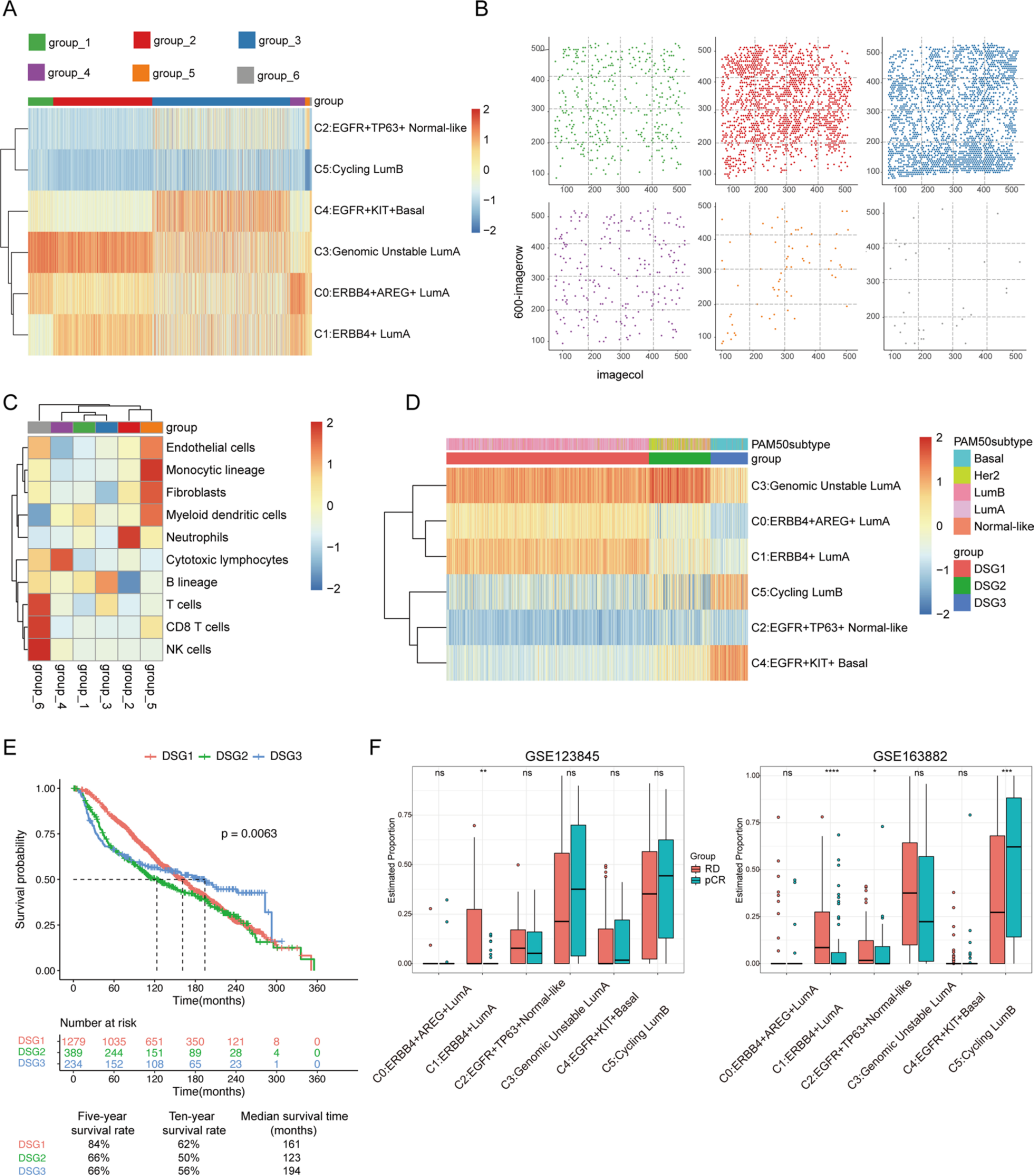
ST analysis of BC-A and cell type deconvolution. **a** Scaled deconvolution values for six epithelial subclusters overlaid onto tissue spots. **b** Different spatial distribution of six groups as defined in **a**. **c** Heatmap of estimated scores of immune cells in each group by MCPcounter. **d** Heatmap of the ssGSEA score of six gene signatures estimated in each METABRIC sample. **e** Kaplan–Meier survival curve for METABRIC cohort in three groups. P value was calculated with log-rank test. Log-rank p value < 0.05 was considered as statistically significant. **f** Box plot of the estimated proportion of six gene signatures in two NAC cohorts using CIBERSORTx. Statistical significance was determined using a two-sided t-test in a pairwise comparison of means between groups, with P values adjusted using the Benjamini–Hochberg procedure. *P < 0.05, **P < 0.005, ***P < 0.005

We deconvoluted breast cancer profiles in the METABRIC cohort [8]. Based on the defined gene signature, all of cases were divided into three discriminated subgroups (DSGs). And DSG2, representing the HER2-overexpressing subtypes based on PAM50 was designated the genomic unstable LumA subtypes (Fig. 2d). For survival analysis, patients representing the DSG2 subtype were associated with the worst survival (Fig. 2e). Through examining public datasets of patients treated with chemotherapy[9], the proportions of the *ERBB4*^+^ LumA cells or *EGFR*^+^*TP63*^+^ normal-like cells were associated with poor response to neoadjuvant chemotherapy (NAC) while the ratio of the cycling LumB cluster significantly increased in patients who response to NAC (Fig. 2f) (Additional file 2: Result S4, Additional file 3).

In summary, we have provided a comprehensive approach to depict the heterogeneity and the architecture of breast cancer. Our study could provide novel insights into the ecosystem of breast cancer and novel therapeutic strategies.

## Supplementary Information

**Additional file 1.** Figures.

**Additional file 2.** Results.

**Additional file 3.** Materials and methods.

## Data Availability

The datasets used and/or analyzed during the current study are available from the corresponding author on reasonable request.

## Abbreviations

snRNA-seq: Single-nucleus RNA sequencing
ST: Spatial transcriptomics
BC: Breast cancer
CNV: Copy number variation
LumA: Luminal-A
LumB: Luminal-B
AREG: Amphiregulin
EGF: Epidermal growth factor
TGFα: Transforming growth factor alpha
EMT: Epithelial–mesenchymal transition
PTN: Pleiotrophin
EFNA5: Ephrin
TFs: Transcription factors
LTF: Lactotransferrin
ECM: Extracellular matrix
H&E: Hematoxylin and eosin
DCIS: Ductal carcinoma in situ
IHC: Immunohistochemistry
ER: Estrogen receptor
PR: Progesterone receptor
DSG: Discriminated subgroups
NAC: Neoadjuvant chemotherapy
pCR: Pathologic complete response
RD: Residual disease
