# Supplementary material for: Single-cell and spatially resolved analysis uncovers cell heterogeneity of breast cancer
Source: J Hematol Oncol. 2022 Mar 3;15:19. doi: 10.1186/s13045-022-01236-0 (PMC8895670; doi:10.1186/s13045-022-01236-0)
Supplement: Supplementary file 1 — Additional file 1. Figures. [file 13045_2022_1236_MOESM1_ESM.pdf]

Supplementary Figures

Fig.S1

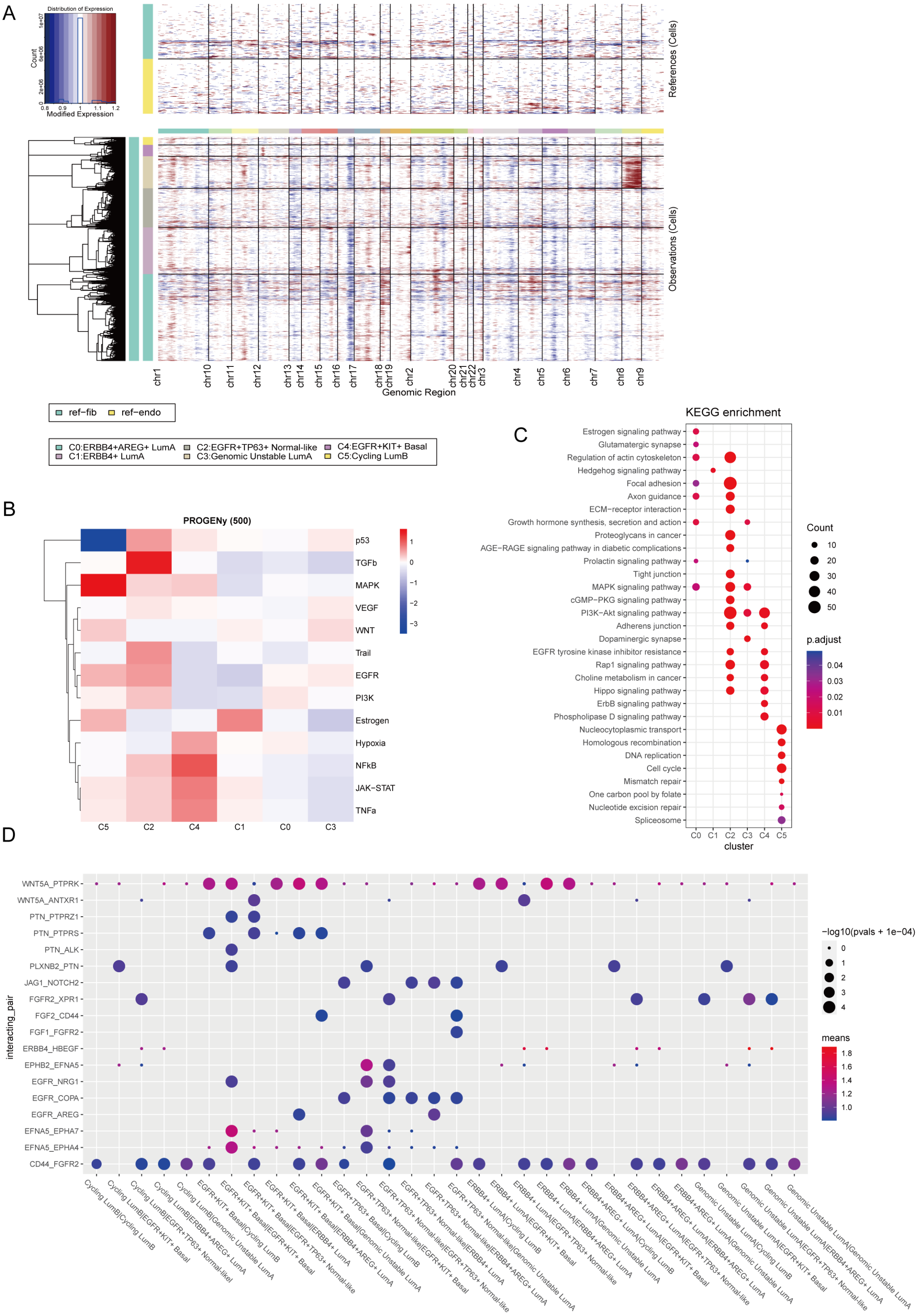

Figure S1 Supplementary figure for snRNA-seq analysis of BC-A. (A) CNV profiles inferred from snRNA-seq on BC-A by inferCNV. Red indicates chromosomal amplifications and blue indicates chromosomal deletions. Fibroblasts and endothelial cells were set as reference. (B) Heatmap showing the pathway enrichment of the each epithelial subcluster using PROGENy. (C) Dot plot showing the pathway enrichment of the each epithelial subcluster using KEGG datasets. (D) Dot plot showing ligand and receptor interaction pairs between epithelial subclusters.

Fig.S2

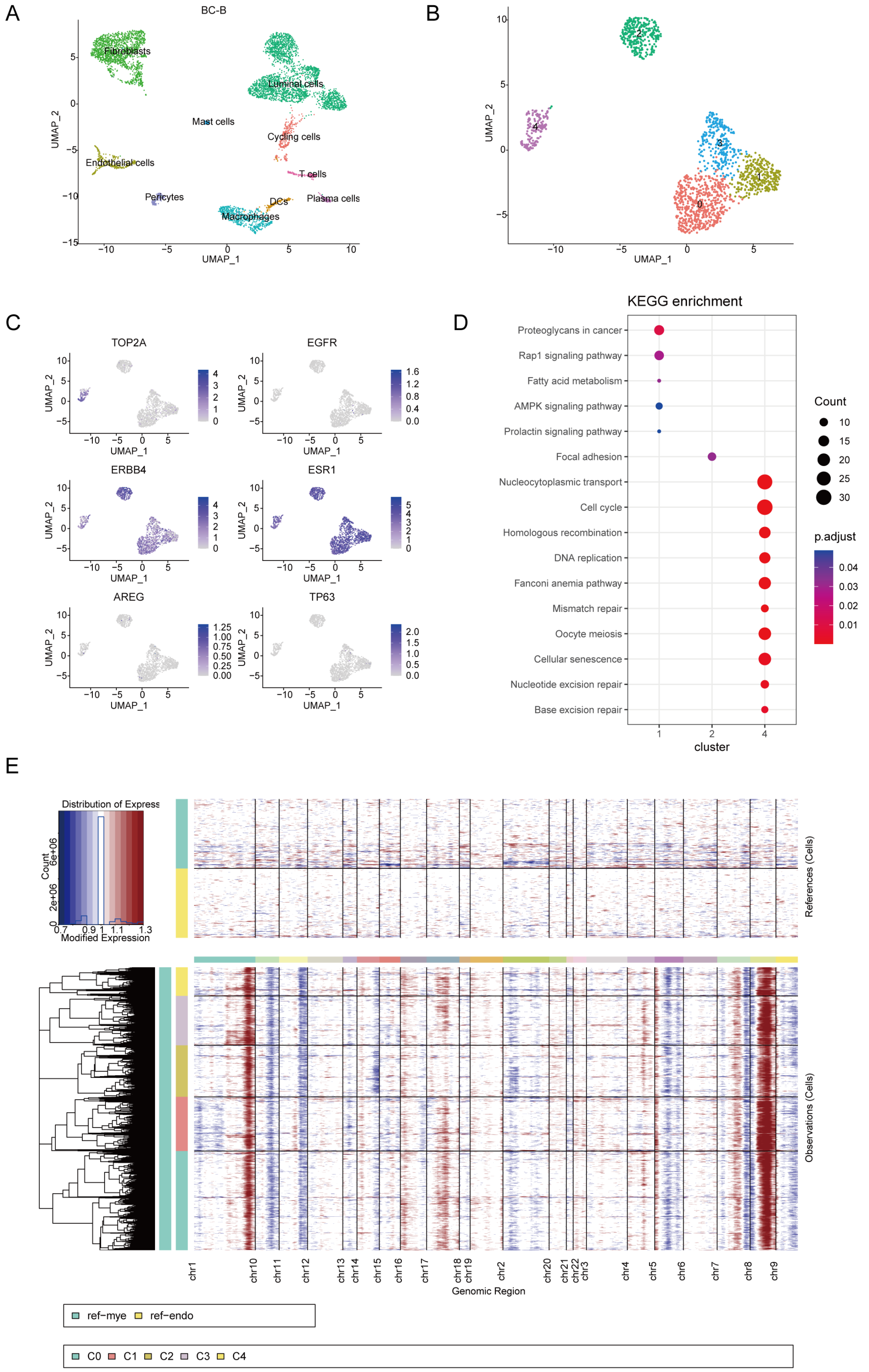
A
BC-B
Fibroblasts
Luminal cells
Mast cells
Cycling cells
Endothelial cells
T cells
Pericytes
DCs
Plasma cells
Macrophages
UMAP_1
UMAP_2
B
C
TOP2A
EGFR
ERBB4
ESR1
AREG
TP63
D
KEGG enrichment
Proteoglycans in cancer
Rap1 signaling pathway
Fatty acid metabolism
AMPK signaling pathway
Prolactin signaling pathway
Focal adhesion
Nucleocytoplasmic transport
Cell cycle
Homologous recombination
DNA replication
Fanconi anemia pathway
Mismatch repair
Oocyte meiosis
Cellular senescence
Nucleotide excision repair
Base excision repair
cluster
Count
p.adjust
E
Distribution of Express
Count
Modified Expression
References (Cells)
Observations (Cells)
Genomic Region
ref−mye
ref−endo
C0
C1
C2
C3
C4

Figure S2 snRNA-seq analysis of tumor from sample BC-B. (A) UMAP visualization of 5917 nuclei from BC-B tumor analyzed by snRNA-seq showing ten major cell types. (B) UMAP visualization of inferred epithelial cells from BC-B tumor analyzed by snRNA-seq. Clusters are colored and labeled according to their inferred cell subtypes. (C) Feature plot of subcluster-specific marker genes in epithelial cells. (D) Dot plot showing the pathway enrichment of the each epithelial subcluster using KEGG datasets. (E) CNV profiles inferred from snRNA-seq on BC-B by inferCNV. Red indicates chromosomal amplifications and blue indicates chromosomal deletions. Fibroblasts and myeloid cells were set as reference.

Fig.S3

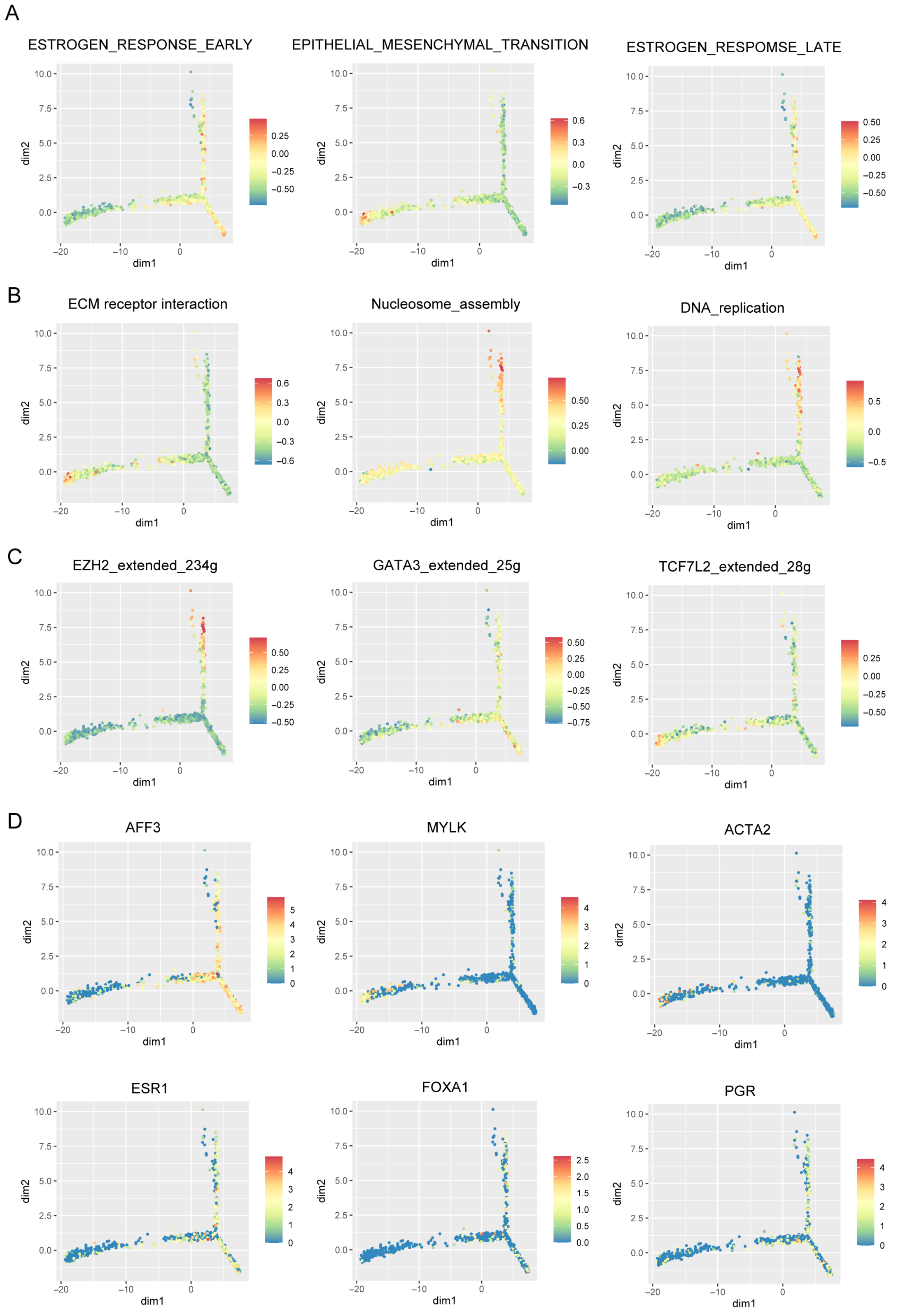
A
ESTROGEN_RESPONSE_EARLY
EPITHELIAL_MESENCHYMAL_TRANSITION
ESTROGEN_RESPOMSE_LATE
B
ECM receptor interaction
Nucleosome_assembly
DNA_replication
C
EZH2_extended_234g
GATA3_extended_25g
TCF7L2_extended_28g
D
AFF3
MYLK
ACTA2
ESR1
FOXA1
PGR
dim1
dim2

Figure S3 Supplementary figure for pseudotime analysis. (A) and (B) Pseudotime trajectory plots showing different pathways enriched in different states. (C) Pseudotime trajectory plots showing different transcription factors enriched in different states. (D) Pseudotime trajectory plots showing genes enriched in different states.

Fig.S4

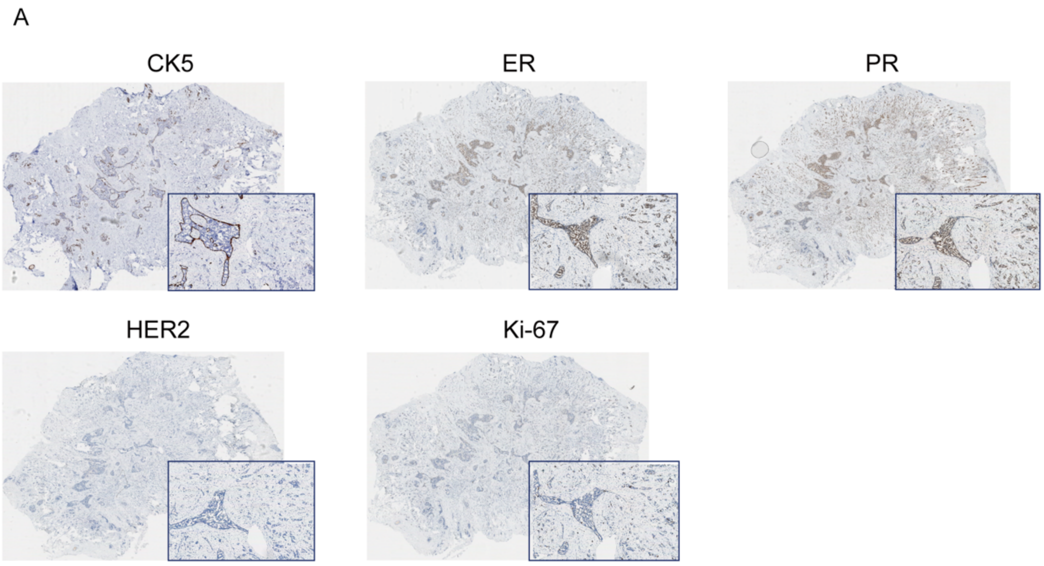

Figure S4 IHC stain validates the histological composition. (A) IHC stain of CK5, ER, PR, Her-2 and Ki-67 from BC-A tumor.

Fig.S5

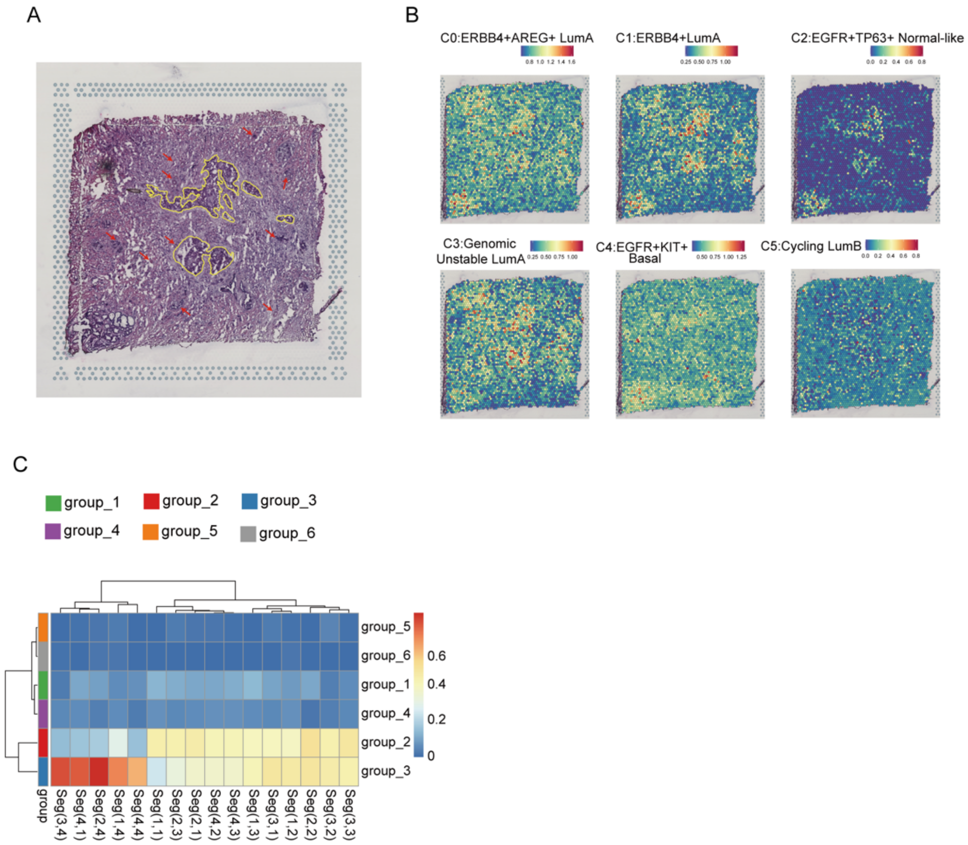

Figure S5 Supplementary figure for cell type deconvolution. (A) Annotated BC-A tumor cryosection on the ST slide. Ductal carcinoma *in situ* (yellow), normal tissue (red). (B) Clustering of the BC-A ST spots in six groups. (C) Heatmap of spot counts of six groups in 16 average areas.

Fig.S6

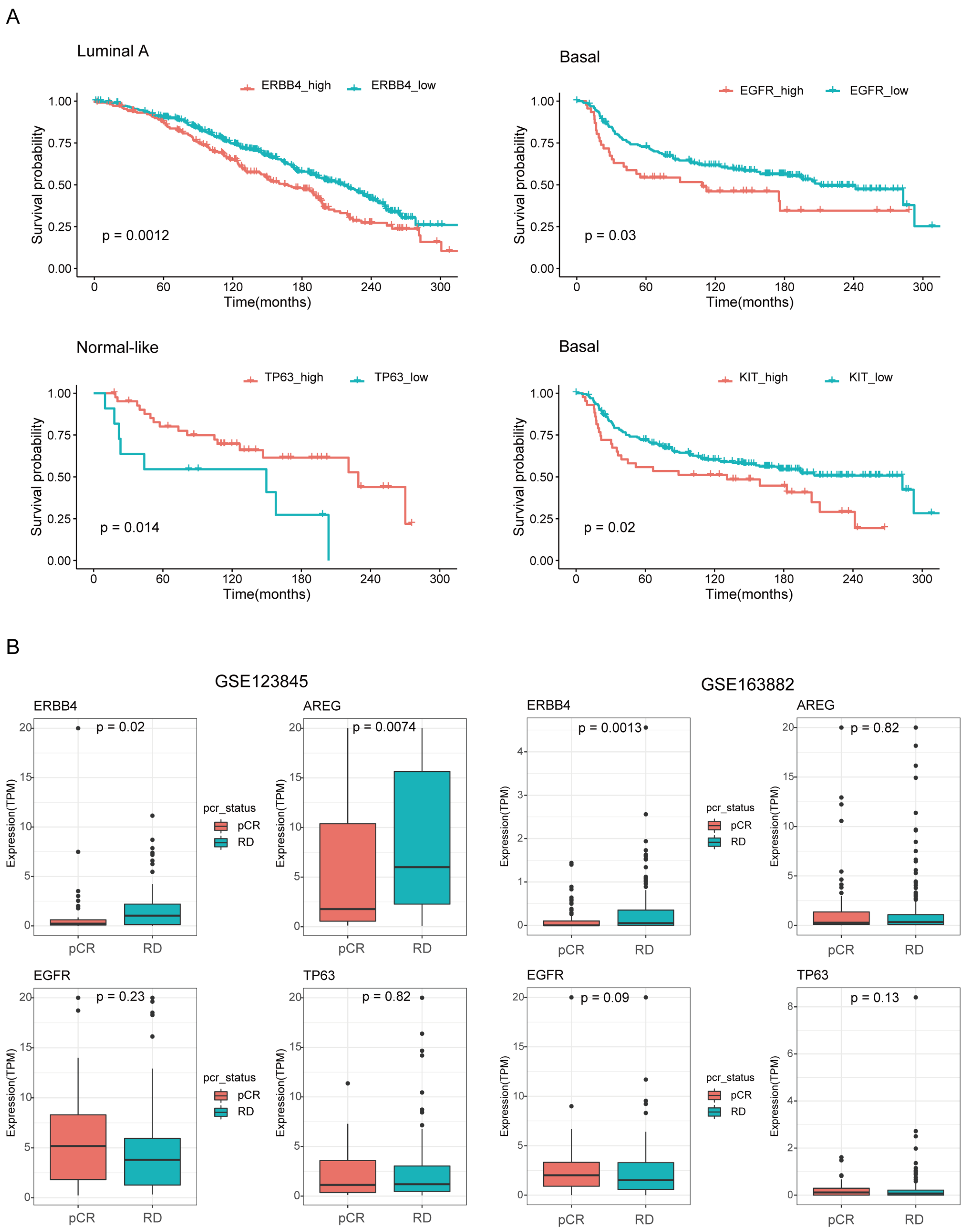

Figure S6 Supplementary figure for survival analysis. (A) Kaplan–Meier survival curve for a specific subtype of METABRIC cohort of gene expression. P value was calculated with log-rank test. Log-rank p value < 0.05 was considered as statistically significant. (B) Boxplot of the expression of *ERBB4, EGFR, AREG, TP63* in the two NAC cohorts. P value was calculated with Wilcoxon test.
