# Supplementary material for: Single-cell and spatially resolved analysis uncovers cell heterogeneity of breast cancer
Source: J Hematol Oncol. 2022 Mar 3;15:19. doi: 10.1186/s13045-022-01236-0 (PMC8895670; doi:10.1186/s13045-022-01236-0)
Supplement: Supplementary file 2 — Additional file 2. Results. [file 13045_2022_1236_MOESM2_ESM.pdf]

Supplementary Results

Result S1 snRNA-seq analysis of tumor from sample BC-B

By analyzing the transcriptomes, we detected 10 distinct cell clusters in the BC-B tumors by using canonical lineage markers (Figure S2A). Notably, remarkable heterogeneity existed between BC-A and BC-B. The epithelial cells were further re-clustered, and 5 subclusters were identified (Figure S2B). The CNV profiles show the amplification on chromosomes 1 and 8 in all subclusters was characterized in the BC-B profile (Figure S2E). Furthermore, the neoplastic clusters in the BC-B sample showed a lack of remarkable biomarkers, only high expression of *TOP2A* was discovered (Figure S2C).

Result S2 Supplementary results for snRNA-seq analysis

Considering the functional characteristics of these subpopulations, *ERBB4*$^+$*AREG*$^+$ LumA cells featured high expression of amphiregulin (AREG) (Figure 1E). AREG is an epidermal growth factor (EGF)-like and transforming growth factor alpha (TGFα)-like ligand which can promote malignant growth[1]. Moreover, this cluster was enriched in Hedgehog signaling, estrogen receptor (ER) and the TGF pathway (Figure 1F). AREG can be stimulated by estrogen and its receptor ER alpha, and it is required for estrogen-induced proliferation[1]. *EGFR*$^+$*TP63*$^+$ normal-like cells were exclusively enriched in WNT signaling, angiogenesis, myogenesis and epithelial-mesenchymal transition (EMT), while *EGFR*$^+$*KIT*$^+$ basal cells were accompanied by enhancement of apoptosis, the P53 pathway and the reactive oxide species pathway (Figure 1F).

Likewise, lactotransferrin (LTF) was particularly enriched in *ERBB4*$^+$ LumA cells, while *EGFR*$^+$*TP63*$^+$ normal-like cells were found to have upregulated activity levels of CREB1/5, TEAD1 and TCF7L2 (a pivotal TF in the WNT signaling pathway) (Figure 1G). In terms of the remaining populations, TEAD1 was enriched in two subclusters, while the cycling LumB cluster showed high RAD21 involved in the repair of DNA double-strand breaks, and EZH2 (a histone methyltransferase subunit of a polycomb

repressor complex) activity (Figure 1G).

In addition, TP63 was identified as an essential regulator of basal/myoepithelial lineage differentiation. In pseudotime analysis, *EGFR*$^{+}$*TP63*$^{+}$ normal-like subtype in the left arm had the activated pathways involved in EMT and extracellular matrix (ECM) receptor interaction potentially regulated by TCF7L2 (Figure 1H, Figure S3A-B). Meanwhile, this state had a tendency to be endowed with some features of luminal tumor clusters. For the potential mechanism, AFF3 (a key TF for ER signaling activation) and GATA3 gradually increased towards luminal states (Figure 1H, Figure S3C-D). The cycling LumB cluster in the arm of the luminal progenitor was enriched in DNA replication and nucleosome assembly pathways by upregulated expression of multiple TFs, such as EZH2 (Figure 1H, Figure S3B-C).

In ligand-receptor crosstalk, EFNA5 encodes a membrane ligand, ephrin A5, whose interaction with EPHA4 and EPHA4 results in an activating signal involved in tumor proliferation and invasion. In contrast, pleiotrophin (PTN) is specifically released by the *EGFR*$^{+}$*KIT*$^{+}$ basal cells, and it is regarded as a heparin-binding growth factor that is conductive to tumorigenesis, angiogenesis and cell migration. Finally, the classical intratumoral exchange mechanisms containing WNT and NOTCH pathways were found to be mainly activated among these subpopulations (Figure 1I, Figure S1D). Additionally, fibroblast growth factors such as FGF1, FGF2 and FGF13, which stimulate malignant growth and metastasis by interacting with their receptors, were overexpressed by some tumorous populations (Figure 1I, Figure S1D).

Result S3 Supplementary results for ST analysis

Spatially resolved transcriptomics was further performed to directly observe the distribution of these neoplastic clusters. In this section, two primary regions contained ductal carcinoma *in situ* (DCIS) and stroma (Figure S5A). Immunohistochemistry (IHC) staining results further demonstrate that the expression of CK5 was appropriately high around the DCIS region and the positive distribution of ER and progesterone receptor (PR) prevailed in DCIS districts (Figure S4). Interestingly, the strength of the *ERBB4*$^{+}$ LumA signatures was mainly located in the DCIS areas. In contrast, the enrichment of

the *EGFR*$^+$*KIT*$^+$ basal subtype was found to disperse across the zone of invasive cancer (Figure S5B). Moreover, the *EGFR*$^+$*KIT*$^+$ basal subcluster was inimitably distributed in the basal region, while the *ERBB4*$^+$*AREG*$^+$ LumA populations were enriched in the infiltrating lymphocyte area accompanied by high infiltration of cytotoxic lymphocytes (Figure 2B-C, S5C). In consideration of the spatial distribution of those nontumor cells in ST, the effector cells containing NK cells and T cells were absent in the cancer region; by contrast, fibroblasts, endothelial cells and myeloid cells were enriched in the stromal area (Figure 2C). Therefore, these diverse tumor subtypes are distributed in mutually autocephalous regions of breast cancers.

Result S4 Supplementary results for cell type deconvolution in bulk transcriptomics

In clinical practice, DSG1 was mainly was predominantly composed of luminal A and luminal B; DSG2 was designated the HER2-overexpressing subtype, while the basal cluster prevailingly aggregated in DSG3 (Figure 2D). Furthermore, the genomic unstable LumA cluster was also focused on the DSG2 gene signature. Likewise, DSG3 was also rich in the cycling LumB cells (Figure 2D).

Survival analyses of the biomarkers were performed in the METABRIC cohort. *ERBB4* was associated with worse survival in the LumA subtype tumors, and high *EGFR* and *KIT* expression level both predicted a worse survival in the basal subtype tumors (Figure S6A). Moreover, we investigated the association between the expression level of the four potential biomarkers and treatment response in two NAC breast cancer cohorts. The expression of *ERBB4* was significantly higher in the NAC non-responding (RD, residual disease) group consistently in the two cohorts. The significance of AREG was ambiguous, while *EGFR* and *TP63* was both neutral (Figure S6B).

Ultimately, we further examined public datasets of patients treated with chemotherapy. We compared the proportions of these malignant clusters in either responders (pCR, pathologic complete response) or non-responders (RD, residual disease), as defined in the original studies [2]. The results indicates that the proportions of the *ERBB4*$^+$ LumA cells were associated with poor response to NAC (Figure 2F). Given that LTF, an age-

related biomarker, is highly expressed in *ERBB4*$^{+}$ LumA cells[3], senescent features endow tumor cells with the capacity for resistance[4]. Therefore, the cycling LumB subtype predicts the susceptibility to treatment, while the subpopulations with overexpression of EGF-associated receptors or aging hallmarks enable resistance chemotherapy.

Supplementary references

[1] C. Berasain, M.A. Avila, Amphiregulin, Semin Cell Dev Biol 28 (2014) 31-41.

[2] Y.H. Park, S. Lal, J.E. Lee, Y.L. Choi, J. Wen, S. Ram, Y. Ding, S.H. Lee, E. Powell, S.K. Lee, J.H. Yu, K.A. Ching, J.Y. Nam, S.W. Kim, S.J. Nam, J.Y. Kim, S.Y. Cho, S. Park, J. Kim, S. Hwang, Y.J. Kim, V. Bonato, D. Fernandez, S. Deng, S. Wang, H. Shin, E.S. Kang, W.Y. Park, P.A. Rejto, J. Bienkowska, Z. Kan, Chemotherapy induces dynamic immune responses in breast cancers that impact treatment outcome, Nat Commun 11(1) (2020) 6175.

[3] A.L. Cardoso, A. Fernandes, J.A. Aguilar-Pimentel, M.H. de Angelis, J.R. Guedes, M.A. Brito, S. Ortolano, G. Pani, S. Athanasopoulou, E.S. Gonos, M. Schosserer, J. Grillari, P. Peterson, B.G. Tuna, S. Dogan, A. Meyer, R. van Os, A.U. Trendelenburg, Towards frailty biomarkers: Candidates from genes and pathways regulated in aging and age-related diseases, Ageing research reviews 47 (2018) 214-277.

[4] E. Mavrogonatou, H. Pratsinis, D. Kletsas, The role of senescence in cancer development, Seminars in cancer biology 62 (2020) 182-191.
