# Supplementary material for: Single-cell and spatially resolved analysis uncovers cell heterogeneity of breast cancer
Source: J Hematol Oncol. 2022 Mar 3;15:19. doi: 10.1186/s13045-022-01236-0 (PMC8895670; doi:10.1186/s13045-022-01236-0)
Supplement: Supplementary file 3 — Additional file 3. Materials and methods. [file 13045_2022_1236_MOESM3_ESM.pdf]

Supplementary Materials and Methods

Clinical specimens

Two primary untreated breast cancer samples (BC-A, BC-B) were used in this study. Samples were analyzed from fresh surgical resections and cryopreserved tissue. BC-A, a 50-year-old female with bilateral breast lumps. Bilateral modified radical mastectomy was performed and the postoperative pathology showed: (Left) invasive ductal breast carcinoma (WHO grade II), ductal carcinoma *in situ* visible around, lymph node metastases, vascular tumor thrombus and Paget's disease of the left nipple. Immunohistochemistry of the tumor was 95% positive for ER, 80% positive PR, 30% positive for Ki67, positive for Her-2, GATA3, E-cadherin, P120, PCK, and negative for AR. (Right) invasive ductal breast carcinoma (WHO grade I-II) with lymph node metastases, nerve invasion, vascular tumor thrombus and para-lymph node adipose tissue carcinoma infiltration. Immunohistochemistry of the tumor was 90% positive for ER, 80% positive for PR and AR, 10%-15% positive for Ki67, positive for Her-2, GATA3, E-cadherin, P120, and negative for Calponin, P63. Immunohistochemistry of the metastasis was 90% positive for ER and PR, 40% positive for AR, 20% positive for Ki67, positive for Her-2, GATA3. BC-B, a female with a right breast lump. Right modified radical mastectomy was performed and the postoperative pathology showed: (Right) invasive ductal carcinoma with lymph node metastases. Immunohistochemistry of the metastasis was 95% positive for ER, 90% positive for PR and AR, 50% positive for Ki-67, and positive for Her-2, GATA3, MUC1.

Nuclei isolation

Nuclei were isolated following a modified nuclear isolation protocol[1]. In brief, frozen human BC tissues were thawed on ice, minced to 1 mm3 and homogenized in cold 0.1% CHAPS in Tris-HCl. The minced adipose tissue was filtered through a 40-μm cell strainer and centrifuged at 500*g* for 5 min at 4 °C, and the pellet was resuspended in

phosphate-buffered saline (PBS) with DAPI. Nuclei suspensions were loaded to a MoFlo Astrios EQ Cell Sorter and sorted into a 1.5-ml tube.

Single-nucleus RNA-sequencing

10X-based libraries were acquired with the Chromium Single Cell V3.0 reagent kit following the manufacturer's protocol (10X Genomics). Nuclei suspensions containing around 500 nuclei per μl were loaded into nine independent lanes. Libraries were sequenced on a NovaSeq 6000 (Illumina)[2].

Single-nucleus RNA-sequencing analysis

The snRNA-seq data from 10X Genomics were aligned and quantified using the Cell Ranger Single-Cell Software. The preliminary filtered data generated from Cell Ranger was used for downstream analysis. Each 10x library was individually quality checked, and cells were filtered to ensure good gene coverage, a consistent range of read counts and low numbers of mitochondrial reads. At least 200 and no more than 5000 detected gene were required for each cell. No more than 15% mitochondrial reads were allowed per cell. Downstream statistical analyses were conducted using the Seurat (V4.0.4) software packages for R.

We processed approximately 8000-9000 cells in each sample. After quality control and filtering, 4,093 single nuclei in the BC-A sample and 5,917 single nuclei in the BC-B sample were retained. UMAP visualization showed major cell types containing fibroblasts, dendritic cells (DCs), endothelial cells, pericytes, macrophages, mast cells, T cells, luminal, and basal tumor cells by using canonical lineage markers.

To distinguish neoplastic cells from normal epithelial cells, single-cell copy number variation (CNV) profiles were estimated by using inferCNV package of R. Fibroblasts and endothelial cells were regarded as the negative control. FindMarkers function of Seurat was used to find differentially expression genes. For calling molecular subtypes using the PAM50 method, we processed 'pseudobulk' expression profiles for each tumor, in a similar manner to any bulk RNA-seq sample. KEGG enrichment and GSVA enrichment were performed using the MSigDB HALLMARK gene sets and the

PROGENy gene sets. SCENIC analysis[3] was performed to measure the difference between cell clusters based on transcription factors or their target genes by using SCENIC package of R. Pseudotime analysis was performed with Monocle2[4] to determine the dramatic translational relationships among cell types and clusters. Cell–cell communication analysis was performed by using CellPhoneDB [5] and iTALK package of R.

Histology and immunohistochemical staining

Tumor tissue was fixed in 10% neutral buffered formalin for 24 h and then processed for paraffin embedding. Diagnostic tumor blocks were accessed for samples that did not have a research block available. Blocks were sectioned at 4 μM. Sections were stained with hematoxylin and eosin (H&E) for standard histological analysis. We marked the distinct histological features in the slide, two primary regions contained ductal carcinoma *in situ* (DCIS) and stroma. IHC was performed on serial sections with prediluted primary antibodies against CK5, ER, PR, HER2 or Ki-67 using suggested protocols on the BOND RX Autostainer (Leica Biosystems). Antigen retrieval was performed for 20 min, followed by primary antibody incubation for 60 min and secondary staining with the Bond Refine Detection System (Leica Biosystems). Slides were imaged using the Aperio CS2 Digital Pathology Slide Scanner and processed with QuPath v.0.2.0.

Spatial transcriptomics

Tissue samples were embedded in optimal cutting temperature compound and stored at −80 °C. Tissue blocks were cut into 10-μm sections and processed using the Visium Spatial Gene Expression Kit (10x Genomics) according to the manufacturer's instructions. First, breast tissue permeabilization condition was optimized using the Visium Spatial Tissue Optimization Kit, which was found to be ideal at 12 min. Sections were stained with H&E and imaged using a Leica DM6000 microscope under a 20× lens magnification, then processed for spatial transcriptomics. The resulting complementary DNA library was checked for quality control, then sequenced using an

Illumina NovaSeq 6000 system. Cycling conditions were set for 28, 98 and 8 for Read 1, Read 2 and Read 3 (i7 index), respectively. Spots were annotated by a specialist breast pathologist using the Loupe v.4.0.0 software (10x Genomics).

Spatial transcriptomics analysis

Reads were demultiplexed and mapped to the reference genome GRCh38 using the Space Ranger software v.1.0.0 (10x Genomics). Count matrices were loaded into Seurat v.3.2.0 and STutility v.0.1.0 for all subsequent data filtering, normalization, filtering, dimensional reduction and visualization. Data normalization was performed on independent tissue sections using the variance-stabilizing transformation method implemented in the SCTransform function in Seurat.

Deconvolution of bulk RNA-seq transcriptomics and spatial transcriptomics

We performed an integrative gene signature consisting of the top 20 differentially expressed genes in each cluster. ssGSEA was used to deconvolute predicted cell fractions from bulk transcript profiling datasets. Normalized METABRIC expression matrices, clinical information and PAM50 subtype classifications were obtained from METABRIC[6]. Tumor subgroups in the METABRIC cohort were identified using SKmeans-based consensus clustering of the predicted cell fraction from ssGSEA in each bulk METABRIC patient tumor.

We performed deconvolution of spatial tissue locations and the scaled deconvolution values for six epithelial subclusters were overlaid onto tissue spots. The ST dataset was classified into distinct regions based on SKmeans-based consensus clustering of the predicted cell fraction from MCPcounter[7]. We predicted the estimated scores of immune cells in each group by using MCPcounter.

Survival analysis of snRNA-seq signatures

To assess the impact of cell types described by snRNA-seq on clinical outcome, we assessed the association between gene signatures with patient overall survival in the METABRIC cohort. Based on the defined gene signatures, all the cases were divided

into three discriminated subgroups (DSGs). Survival curves of the three groups were generated using the Kaplan–Meier method with the 'survival' package. We assessed the significance between each two groups using the log-rank test statistics. R package 'survminer' was used for visualization.

Chemotherapy resistance analysis

We examined public datasets of patients treated with chemotherapy. Normalized expression matrices and clinical information were obtained from GEO under accession number GSE123845 and GSE163882. CIBERSORTx[8] was used to predict the estimated scores of each gene signatures in two NAC cohorts. Statistical significance was determined using a two-sided t-test in a pairwise comparison of means between groups, with P values adjusted using the Benjamini–Hochberg procedure.

Supplementary references

[1] E. Drokhlyansky, C.S. Smillie, N. Van Wittenberghe, M. Ericsson, G.K. Griffin, G. Eraslan, D. Dionne, M.S. Cuoco, M.N. Goder-Reiser, T. Sharova, O. Kuksenko, A.J. Aguirre, G.M. Boland, D. Graham, O. Rozenblatt-Rosen, R.J. Xavier, A. Regev, The Human and Mouse Enteric Nervous System at Single-Cell Resolution, Cell 182(6) (2020) 1606-1622 e23.

[2] W. Sun, H. Dong, M. Balaz, M. Slyper, E. Drokhlyansky, G. Colleluori, A. Giordano, Z. Kovanicova, P. Stefanicka, L. Balazova, L. Ding, A.S. Husted, G. Rudofsky, J. Ukropec, S. Cinti, T.W. Schwartz, A. Regev, C. Wolfrum, snRNA-seq reveals a subpopulation of adipocytes that regulates thermogenesis, Nature 587(7832) (2020) 98-102.

[3] S. Aibar, C.B. Gonzalez-Blas, T. Moerman, V.A. Huynh-Thu, H. Imrichova, G. Hulselmans, F. Rambow, J.C. Marine, P. Geurts, J. Aerts, J. van den Oord, Z.K. Atak, J. Wouters, S. Aerts, SCENIC: single-cell regulatory network inference and clustering, Nat Methods 14(11) (2017) 1083-1086.

[4] X. Qiu, Q. Mao, Y. Tang, L. Wang, R. Chawla, H.A. Pliner, C. Trapnell, Reversed

graph embedding resolves complex single-cell trajectories, Nat Methods 14(10) (2017) 979-982.
[5] M. Efremova, M. Vento-Tormo, S.A. Teichmann, R. Vento-Tormo, CellPhoneDB: inferring cell-cell communication from combined expression of multi-subunit ligand-receptor complexes, Nat Protoc 15(4) (2020) 1484-1506.
[6] C. Curtis, S.P. Shah, S.F. Chin, G. Turashvili, O.M. Rueda, M.J. Dunning, D. Speed, A.G. Lynch, S. Samarajiwa, Y. Yuan, S. Graf, G. Ha, G. Haffari, A. Bashashati, R. Russell, S. McKinney, M. Group, A. Langerod, A. Green, E. Provenzano, G. Wishart, S. Pinder, P. Watson, F. Markowetz, L. Murphy, I. Ellis, A. Purushotham, A.L. Borresen-Dale, J.D. Brenton, S. Tavare, C. Caldas, S. Aparicio, The genomic and transcriptomic architecture of 2,000 breast tumours reveals novel subgroups, Nature 486(7403) (2012) 346-52.
[7] E. Becht, N.A. Giraldo, L. Lacroix, B. Buttard, N. Elarouci, F. Petitprez, J. Selves, P. Laurent-Puig, C. Sautes-Fridman, W.H. Fridman, A. de Reynies, Estimating the population abundance of tissue-infiltrating immune and stromal cell populations using gene expression, Genome Biol 17(1) (2016) 218.
[8] A.M. Newman, C.B. Steen, C.L. Liu, A.J. Gentles, A.A. Chaudhuri, F. Scherer, M.S. Khodadoust, M.S. Esfahani, B.A. Luca, D. Steiner, M. Diehn, A.A. Alizadeh, Determining cell type abundance and expression from bulk tissues with digital cytometry, Nat Biotechnol 37(7) (2019) 773-782.
